# Epidemiology of drowning clients in Shiraz Emergency Medical Service (EMS) in 2020 

**Published:** 2022-01

**Authors:** Mohammad Javad Moradian, Behnaz Rastegarfar, Farahnaz Fooladband

**Affiliations:** ^*a*^ Assistant Professor, Department of Health in Emergencies and Disasters, Faculty of Management and Medical Informatics, Shiraz University of Medical Sciences, Shiraz, Iran.; ^*b*^ Ph.D. in Disaster and Emergency Health, Department of Disaster and Emergency Health, Faculty of Public Health, Tehran University of Medical Sciences, Tehran, Iran.; ^*c*^ Quality Improvement Expert, Disaster and Emergency Medical Management Center, Shiraz University of Medical Sciences, Shiraz, Iran.

## Abstract

**Background:**

Drowning is one of the health problems in the world and one of the top ten causes of traumatic deaths in Iran. Managers' awareness of the characteristics of drowning clients helps to formulate and implement an intervention plan. The aim of this study was to investigate the epidemiology of drowning clients in Shiraz Emergency Medical Service (EMS) from 2019 to 2020.

**Methods:**

Using the census method, the names of 62 people (all drowning emergency clients) and their information (age, sex, time and place of drowning, and the result of the emergency team mission) were extracted from Shiraz EMS in the years 2019-2020 and analyzed using SPSS (Version 23).

**Results:**

Of all EMS clients, 0.02% were emergency drowning cases; 70.2% of them were male and 29.8% were female. Their mean age was 20.5 years (29.8% of children, 15.8% adolescents, 33.4% youth, 17.5% middle-aged, and 3.5% the elderly). About half of the drownings (47.4%) happened in the spring and 31.6% in the summer. More than half of the drownings (56.1%) occurred at 11 AM-3 PM and 32.3% at 4 PM-8 PM. Regarding the outcome of drowning, 42.1% of the drowned died, 42.1% were admitted to the hospital, 7% were cared at the scene and 8.8% of them did not cooperate to be transported to the hospital. Moreover, 42.9% of them had concomitant trauma with drowning, 54.2% of whom had head trauma, 12.5% had shoulder and back trauma and the rest had multiple trauma.

**Conclusions:**

Public awareness should be raised on the possible dangers of drowning through health and media. EMS managers should emphasize considering traumas with drowning by technicians in the EMS. Public education about the EMS duty should be enhanced to gain more trust, participation, and cooperation of clients. Protection and safety enhancement of public pools, ponds, domestic and lakes, etc. should be emphasized by the authorities.

**Keywords:**

Epidemiology; Trauma; Drowning; EMS

